# Therapeutic Antibodies against Intracellular Tumor Antigens

**DOI:** 10.3389/fimmu.2017.01001

**Published:** 2017-08-18

**Authors:** Iva Trenevska, Demin Li, Alison H. Banham

**Affiliations:** ^1^Nuffield Division of Clinical Laboratory Sciences, Radcliffe Department of Medicine, University of Oxford, John Radcliffe Hospital, Oxford, United Kingdom

**Keywords:** T-cell receptor mimic antibody, intracellular antibody, intrabody, MHC class I presented peptide, T-cell epitope, cancer immunotherapy, therapeutic antibody, T-cell receptor-like antibody

## Abstract

Monoclonal antibodies are among the most clinically effective drugs used to treat cancer. However, their target repertoire is limited as there are relatively few tumor-specific or tumor-associated cell surface or soluble antigens. Intracellular molecules represent nearly half of the human proteome and provide an untapped reservoir of potential therapeutic targets. Antibodies have been developed to target externalized antigens, have also been engineered to enter into cells or may be expressed intracellularly with the aim of binding intracellular antigens. Furthermore, intracellular proteins can be degraded by the proteasome into short, commonly 8–10 amino acid long, peptides that are presented on the cell surface in the context of major histocompatibility complex class I (MHC-I) molecules. These tumor-associated peptide–MHC-I complexes can then be targeted by antibodies known as T-cell receptor mimic (TCRm) or T-cell receptor (TCR)-like antibodies, which recognize epitopes comprising both the peptide and the MHC-I molecule, similar to the recognition of such complexes by the TCR on T cells. Advances in the production of TCRm antibodies have enabled the generation of multiple TCRm antibodies, which have been tested *in vitro* and *in vivo*, expanding our understanding of their mechanisms of action and the importance of target epitope selection and expression. This review will summarize multiple approaches to targeting intracellular antigens with therapeutic antibodies, in particular describing the production and characterization of TCRm antibodies, the factors influencing their target identification, their advantages and disadvantages in the context of TCR therapies, and the potential to advance TCRm-based therapies into the clinic.

## Introduction

Historically the consensus in the immunotherapy field has been that antibody therapy is amenable to targeting only extracellular antigens that are accessible for antibody binding. This is due to the fact that the high molecular weight of antibodies prevents them from crossing the cell membrane to access intracellular targets. Consistent with this train of thought, the targets of approved antibody therapies are predominantly extracellular antigens (1). By contrast, small molecules have been used to target those intracellular antigens with a functionality that is suitable for drug screening. In comparison to antibodies, small molecules tend not to be as selective for their targets. They can exhibit unpredictable off-target activities, which consequently lead to adverse side effects and may require a more individualized clinical development pipeline.

More recently, there are three broad approaches whereby antibodies have been used to target intracellular antigens.

(1) It is possible for antibodies (or their derivatives) to target antigens that are normally intracellular but become externalized (for example, during disease).
(2) It is also possible to engineer antibodies or antibody fragments that penetrate into cells, or those that are directly expressed within cells using a gene therapy style approach.
(3) Antibodies can also be generated that bind cell surface major histocompatibility complex class I (MHC-I)-presented peptides that are derived from intracellular proteins.

With further developments in this field, it is becoming clear that the dichotomy between the antibody targeting of intracellular and extracellular targets is not as rigid as originally thought. Antibodies with novel mechanisms of action are challenging this belief and are re-defining the selection of suitable targets for antibody therapy. Antibodies that target intracellular antigens could open the door to a whole new realm of therapeutic targets, with potentially immense clinical benefits. While antibodies targeting intracellular antigens have broad clinical potential, this review will focus primarily on their application for cancer therapy.

## Antibodies Targeting Externalized Antigens

Intracellular antigens can become externalized on the cell surface or secreted and can, therefore, be targeted by antibodies. The Zeng group has further explored the possibility of developing antibodies to intracellular oncoproteins. After an initial proof-of-concept study investigating intracellular proteins targeted by both antibody and vaccine therapy, they focused on phosphatase of regenerating liver 3 (PRL-3) and developed a humanized anti-PRL-3 antibody (2, 3). PRL-3 is a cancer-related phosphatase (4) that is reported to be involved in malignant transformation and metastasis, as well as its expression correlating with poor prognosis (5). It is undetectable in most normal human tissues, is involved in colorectal cancer and uveal melanoma, and is overexpressed in 85% of gastric cancers (but not patient-matched normal gastric tissue), which is the cancer model that has been further studied (3). Importantly, intracellular PRL-3 can be externalized by tumor cells, thus enabling its targeting using classical antibody technology.

It is not the first time that secreted or externalized intracellular proteins have been observed on cancer cells or within the tumor microenvironment and identified as potential therapeutic targets. One such example is the intracellular melanosomal membrane glycoprotein, gp75, which is normally expressed in the melanosome, a specialized organelle present in melanocytes. In melanoma, gp75 is expressed on the cell surface of malignant melanocytes and can be targeted by antibodies in mouse melanoma models (6). In addition, heat-shock proteins 70 and 90 are chaperone proteins, which are further examples of targets that are intracellular in normal cells but become presented on the cell surface, or secreted into the extracellular environment, in transformed cells (7, 8). Tumor cells have been previously shown to shed intracellular material into the tumor microenvironment and extracellular space. This is believed to be a consequence of the inflammatory reaction that surrounds tumor tissues, where immune surveillance can provoke apoptosis and necrosis of tumor cells, thus releasing intracellular components into the extracellular space (9). It has also been suggested that typically intracellular antigens can also be externalized through unconventional secretion pathways (10). This is corroborated by the observation that antibodies against gp75 can reject tumors where there is no necrosis, suggesting an alternative pathway enabling antigen externalization (6). It is the restricted expression profile and the secretion and externalization of PRL-3, by cancer cells, that make it possible to selectively target this oncoprotein with antibody therapy. In this context, it is possible to target an intracellular oncoprotein, which has become externalized onto the cell surface, with an antibody in the same manner as targeting a classical cell surface target.

Several observations have been made on the possible mechanisms of action that mediate the therapeutic effect of targeting extracellularized antigens with a non-neutralizing antibody. It is postulated that, *in vivo*, the Fc portion of these antibodies can be recognized by immune effector cells that have immunoglobulin (Ig) receptors (FcRs), such as macrophages, B cells, and natural killer (NK) cells (11). Therefore, the mechanisms of action could involve a combination of the following:

(1) antibody-dependent cell-mediated cytotoxicity (ADCC) by NK cells,
(2) antibody-dependent cellular phagocytosis by macrophages,
(3) secreted antigens bound to antibody can form immune complexes that can be processed by dendritic cells, which then proceed to activate NK cells (12).

The importance of immune effector cells to the therapeutic efficacy and the aforementioned hypotheses are corroborated by the Zeng group’s previous findings, which showed that anti-PRL-3 antibodies have no therapeutic activity in immunocompromised SCID mice or *in vitro* against PRL-3-expressing cancer cells where no effector cells are present (2, 13). Such engagement with innate immune effectors is a common mechanism of action of therapeutic antibodies that do not modify the activity of the target antigen, including those against cell surface targets.

## Intracellular Antibodies

Intracellular antibodies, which may also be called intrabodies, are antibodies that are produced in the cell, and bind an antigen within the same cell. This is a different delivery strategy from antibodies that are produced extracellularly, and are engineered to then penetrate the cell to access their intracellular target.

Antibodies are soluble proteins that are normally found circulating the body within the serum. They are synthesized in the endoplasmic reticulum (ER) of B cells as separate heavy chain and light chains, which are then linked by disulfide bonds in the mature Ig. However, the full-length antibody is not functional in the cytosol, prior to secretion, due to its reducing conditions, which affect protein folding and the intramolecular disulfide bonds that are required to maintain the antibody’s conformation and stability (14). Fortunately, the complementarity-determining regions that endow an antibody with its exceptional target specificity are located in the variable regions of both the heavy and light chains. Therefore, it is possible to use antibody fragments incorporating the specificity-providing regions within a single-chain variable fragment (scFv), which can be further engineered for cytosolic stability, to target intracellular antigens (15, 16). The scFv is a single polypeptide, which is a favorable characteristic for *in vivo* expression, and it has been studied as a therapeutic for viral infections and cancer, among other diseases.

Furthermore, the variable (V) region domain can be used by itself to form a domain antibody or Dab (17). These can be engineered from conventional human Igs, or also from those from camelids (camel or llama) and cartilaginous fish (carpet or nurse sharks), whose immune systems were found to have evolved high-affinity V-like domains fused to a conserved framework that is reflective of the constant Fc region found in human Ig (18, 19). It has been reported that single heavy chain V regions or light chain V regions can be expressed inside cells. These are referred to as intracellular domain antibodies, which do not require intramolecular disulfide bonds for stability, hence representing the smallest format of the antibody that retains target specificity while minimizing size—a crucial factor for intracellular targeting (20).

There are several critical aspects to generating functional intracellular antibodies. The first is designing an antibody format that will retain its stability and antibody binding capacity within the cell and the second is the ability to introduce or express the antibody within cells. Furthermore, as intracellular antibody fragments do not possess an Fc region (and full length intracellular antibodies cannot recruit extracellular immune effector cells from within the cell), different strategies must be employed to equip them with effector functions unless they have directly neutralizing activity against the target. Examples include ER targeting to cause degradation of the target protein, antibody–antigen interaction-dependent apoptosis that is used to induce programmed cell death through the activation of caspases, and suicide intrabody technology that causes proteolysis of the target protein (21, 22).

In cancer, some of the proteins that are key players in signaling pathways leading to malignant transformation have thus far been inaccessible to small molecule inhibitors (23). In particular, some of these are large, intracellular proteins that act as molecular scaffolds and function primarily through facilitating protein–protein interactions (PPIs). Due to their size, small molecules cannot physically block the large surface of such proteins, nor interfere in the protein–protein interfaces they form, which are typically hydrophobic, flat surfaces, presenting few possibilities for small molecule anchorage (24). This is where technologies that enable the use of antibodies within the cell can bridge the gap between small molecule inhibitors and large protein targets. In this context, the proteins themselves are not the target, but it is the interactions they form with other proteins or nucleic acids that are the therapeutic targets as they contribute to the diseased state. One example is the use of an anti-RAS intrabody, which is composed of a single variable heavy region domain that targets activated GTP-bound RAS. This antibody competitively blocks RAS-effector functions within the tumor cell and while able to prevent *in vivo* tumor initiation and further tumor growth in murine models, it was not curative (25, 26). Thus, some antibodies may enable control of tumor growth and require combinations with additional agents to potentially achieve a cure.

Intrabodies can also be used to characterize the expression of their target proteins and study the *in vivo* knockdown of protein function, and can represent an alternative to generating gene knockout animal models. There are different types of intrabodies that can be tailored to target proteins within subcellular compartments, primarily the cytoplasm or the ER, but the addition of a signal peptide also allows targeting to the mitochondria or the nucleus. This can be used to confer additional subcellular specificity on their intracellular targeting. Importantly, antibodies retained in the ER do not experience the problems with conformation that are caused by the reducing conditions within the cytosol and can be active without neutralizing function against their target (27). For example, using intrabodies targeted to the ER (using a “KDEL” or “SEKDEL” sequence) allows the knockdown of proteins that are passing through the ER, thus abrogating their downstream function in a similar way to RNA interference and providing an alternative strategy for silencing gene products. It has also been proposed that ER-targeting intrabodies may maintain silencing more effectively than short interfering RNA (siRNA) and their specificity may be easier to predict than the off-target effects of an siRNA. An intradiabody that simultaneously enabled the knockdown of VEGF-R2 and Tie-2 was able to reduce both tumor growth and angiogenesis *in vivo* (28). Intrabody technology is overviewed in depth by recent reviews including Marschall and Dübel (29).

### Delivery of Antibodies to the Intracellular Compartment

**Figure 1:**
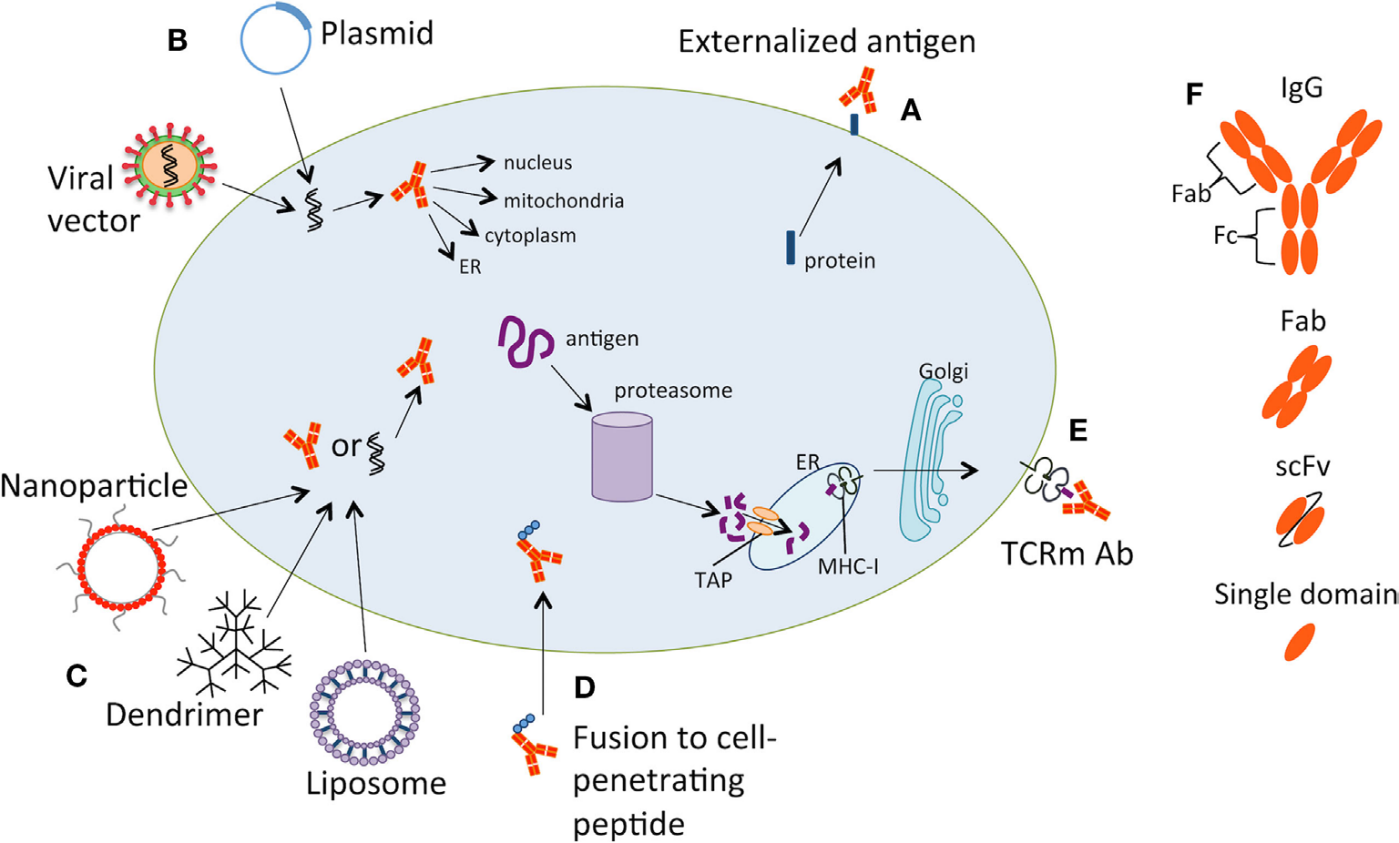
Strategies for targeting intracellular tumor antigens with antibody therapy. Some of the methods for targeting intracellular tumor antigens are illustrated. (A) Intracellular antigens can be externalized on the cell surface or secreted, allowing targeting by antibodies. (B) Plasmids or viral vectors can be used to deliver antibody-encoding genes into the cell. Once internalized, the DNA is transcribed into the targeting antibody, which can be designed to translocate to the nucleus, mitochondria, endoplasmic reticulum (ER), or cytoplasm. (C) Nanoparticles, dendrimers, or liposomes can be used to deliver an antibody or an expression vector encoding the intracellular antibody into the target cell. (D) Antibodies can be fused to cell-penetrating peptides, which allow internalization of the antibody. (E) T-cell receptor mimic (TCRm) antibodies can be used to target peptides bound to major histocompatibility complex class I (MHC-I) molecules on the cell surface. The peptides are derived from intracellular proteins, which have been degraded by the proteasome into short peptides. Peptides are loaded onto MHC-I molecules in the ER, transported through the Golgi apparatus, and finally presented on the cell surface. (F) The antibody depicted on the diagram could represent a full-length IgG, a Fab fragment, scFv or a single domain antibody.

Despite the general consensus that antibodies can only be used to target extracellular or secreted antigens, the cellular uptake of antibodies (by processes such as endocytosis) has been observed both clinically and experimentally in the case of autoimmune disease. It has been reported that once autoantibodies bind their intracellular target, they can cause apoptosis of the cell (30–32). Therefore, the idea of using intracellular antibodies therapeutically represents a logical expansion of such observations. At present, the use of intracellular antibodies is still limited by the technology needed for antibody delivery and they are used primarily as research tools. A number of different methods are being investigated for the delivery of antibodies to the intracellular compartment within target cells. Some of these strategies are illustrated in Figure 1 and they fall into two broad strategies:

(1) The first is a type of “gene therapy” approach using vectors that enable expression of the intracellular antibodies within the target cell—these can be either viral vectors or plasmids.
(2) The second is direct administration of the antibody-based therapeutic—either alone, using electroporation or with dendrimers, liposomes, nanoparticles, or by fusing the antibody to protein-transduction domains that enable it to penetrate the cell (33).

Viral vectors that can be used to deliver the genetic information for expression of intracellular antibodies include adenovirus, adeno-associated virus (AAV), and retrovirus (including lentivirus), which have all been studied extensively in the pre-clinical setting as gene therapy delivery vehicles (34). Retroviruses integrate the antibody fragment expression cassette into the host genome, allowing long-term expression of the intracellular antibody fragment. Despite this advantage, a safety concern with the use of lentiviruses is the risk of integration of the expression cassette in the proximity of an oncogene in the host genome, thereby triggering secondary cancers. By contrast, AAV releases the DNA as an episome, avoiding such safety concerns, however, there is always the possibility of loss of expression, which means relatively shorter term expression.

A non-viral strategy for delivering genes or proteins to the intracellular compartment involves the encapsulation of DNA or proteins in cationic lipid structures called liposomes (35–37). Liposomes form a closed, spherical particle that is amphiphilic and composed of one or more lipid bilayers with an aqueous center. In addition to delivering antibodies, they can also be coated with antibodies that bind cell surface proteins on the target cells (38). Thus, they are targeted to a specific cell type and can deliver an antibody, or an expression vector encoding the intracellular antibody, to the target cell without employing a viral delivery method. Liposomes are internalized *via* endocytosis following interaction with the plasma membrane, which is based on multiple factors, including particle size and charge interactions (39). Nanoparticles are an alternative non-viral method for delivering DNA or antibodies intracellularly (40, 41). They are made of polymers such as poly lactic-co-glycolic acid (PLGA), which is an FDA-approved polymer that has been studied extensively for therapeutic applications (42). PLGA-based nanoparticles have been used to improve the endocytic cellular uptake of antibody fragments such as 3D8 scFv (43). Similarly, antibody-coupled delivery can be used, wherein the expression vector DNA is coupled to the C-terminus of an antibody that binds a cell surface target. The vector DNA is internalized upon internalization of the delivery antibody, and the therapeutic antibody it encodes is then expressed intracellularly (44). Expression vectors and antibodies can also be conjugated to dendrimers (synthetic polymers with a branching tree-like structure) for delivery into target cells (45).

Fusion to cell-penetrating peptides may be an alternative method for delivering antibody fragments into cells through protein transduction. Antibodies that have cell-penetrating peptides fused to them can be referred to as TransMabs (46). The first TransMab that was generated was composed of an anti-caspase-3 antibody fused to a 17 amino acid peptide that could translocate the antibody across the plasma membrane of target cells (47). This then blocked events related to apoptosis, such as caspase-3 activity and DNA fragmentation. Using this method, the antibody–peptide fusion protein enters the cell through endocytosis (48). However, it is difficult to predict whether sufficient macrodrug will enter the cell in order for it to mediate a therapeutic effect. Particularly as cell-penetrating peptides fused to macromolecules have been reported to be at risk of being trapped within endosomes (49). Another disadvantage of this method is that antibody fragments will undergo degradation in the intracellular compartment, as would any protein, therefore, continuous re-administration would be required to maintain any therapeutic activity. A cell-penetrating IgG1 antibody targeting activated GTP-bound RAS (RT11) has recently been shown to block oncogenic signaling and inhibit tumor growth in mouse xenograft models with mutated but not wild type Ras. This iMab (internalizing and PPI interfering monoclonal antibody) has successfully blocked the activity of a highly desirable oncogenic target that lacks effective small molecule inhibitors (50).

## T-Cell Receptor Mimic (TCRm) Antibodies

Immunotherapies targeting intracellular proteins can also exploit the immune system’s own intracellular surveillance mechanism. Intracellular proteins are degraded by the proteasome to form short peptides of specific lengths. These peptides are then presented on the cell surface of most nucleated cells, in a complex with MHC-I molecules (51). CD8^+^ T cells recognize peptide–MHC-I complexes through their clonotypic T-cell receptor (TCR) and become activated to kill malignant or virus-infected cells that present tumor or viral peptides (51). Significantly these MHC-presented peptides do not have a functionality that would make them suitable targets for small molecule drug screening.

Antibodies targeting disease-associated peptide–MHC-I complexes, the so-called TCRm antibodies or TCR-like antibodies, are similar to the TCR in that they bind both the peptide and the MHC-I molecule and, therefore, their binding is both peptide-specific and MHC-restricted (52, 53). TCRm antibodies have expanded the range of targetable antigens to include intracellular proteins without the delivery complications associated with intracellular antibodies. Another advantage of TCRm antibodies is that they combine the intricate tumor specificity of TCRs with the biological properties of antibodies, which do not succumb to immune regulatory mechanisms that obstruct T-cell function in the tumor microenvironment (54). Like conventional monoclonal antibodies, TCRm antibodies have been shown to cause tumor killing through antibody-dependent mechanisms such as cell-mediated cytotoxicity (ADCC) and complement-dependent cytotoxicity (CDC) (55, 56). Furthermore, studies have shown a TCRm antibody to cause apoptosis in breast cancer cells through a caspase-dependent pathway (55). In addition to the success of using naked TCRm antibodies, there have also been reports of anti-tumor activity when they are conjugated to toxins (57, 58). The ability of TCRm Abs to target intracellular antigens has also been applied to cellular therapies in the development of chimeric antigen receptor T cells (59, 60).

### TCRm Antibodies Published to Date

Since the advent of the necessary techniques and technologies, there has been an increase in the production of TCRm antibodies and constructs derived from them. The target peptides of such reagents have typically derived from either viral antigens (including HIV and Hepatitis B antigens) or cancer antigens, and they are commonly presented by either the HLA-A*0201 or the HLA-A*2402 MHC-I haplotype (61, 62). While TCRm antibodies can be used for therapeutic purposes, they are also widely used as research tools for the study of antigen presentation and recognition, as well as for structural studies. Some of the TCRm antibody therapeutics have shown promise in both *in vitro* and *in vivo* studies, however, none of them have advanced to clinical studies. Information on the TCRm and TCR-like antibodies generated to date is summarized in Table 1.

**Table 1:** TCRm antibodies for cancer immunotherapy.

Target	Epitope sequence	MHC haplotype	TCRm antibody name	Isotype/format	Cancer indications investigated	Isolation method	Reference
MAGEA1	EADPTGHSY	HLA-A*0101	Fab-G8	Fab	Melanoma	Phage	(63)
MAGEA1	EADPTGHSY	HLA-A*0101	Fab-Hyb3	Fab	Melanoma	Phage	(64)
GP100	KTWGQYWQV	HLA-A*0201	G2D12, G3G4	Fab	Melanoma	Phage	(65, 66)
GP100	IMDQVPFSV	HLA-A*0201	1A9, 1C8, 1A11, 1A7	Fab	Melanoma	Phage	(65, 66)
GP100	YLEPGPVTV/A	HLA-A*0201	2F1, 2B2, 2C5, 2D1	Fab	Melanoma	Phage	(65, 66)
GP100	IMDQVPFSV	HLA-A*0201	G1	scFv-PE38	Melanoma	Phage	(57)
GP100	ITDQVPFSV	HLA-A*0201	GPA7	sdAb-CAR	Melanoma	Phage	(60)
hTERT	ILAKFLHWL	HLA-A*0201	4A9, 4G9	Fab	Melanoma, prostate	Phage	(67)
hTERT	RLVDDFLLV	HLA-A*0201	3H2, 3G3	Fab	Melanoma, prostate	Phage	(67)
MUC1	LLLTVLTVV	HLA-A*0201	M2B1, M2F5, M3A1, M3B8, M3C8	Fab	Breast	Phage	(68)
NY-ESO-1	SLIMWITQC	HLA-A*0201	3M4E5	Fab	Melanoma	Phage	(69)
MAGE3	FLWGPRALV	HLA-A*0201	7D4, 8A11, 2G12, 9E6	–	–	Hybridoma	(70)
hCGβ	GVLPALPQV	HLA-A*0201	RL4B/3.2G1	mIgG2a	Ovarian, colon, breast	Hybridoma	(71)
hCGβ	GVLPALPQV	HLA-A*0201	1B10	IgG1	Ovarian, colon, breast	Hybridoma	(72)
hCGβ	TMTRVLQGV	HLA-A*0201	3F9	IgG1	Ovarian, colon, breast	Hybridoma	(72)
Her2/Neu	KIFGSLAFL	HLA-A*0201	1B8	IgG1	Breast, colon	Hybridoma	(73)
Melan-A/MART-1	EAAGIGILTV/ELA	HLA-A*0201		Fab	Melanoma	Phage	(74)
Melan-A/MART-1	EAAGIGILTV	HLA-A*0201	CAG10, CLA12	Fab-PE38	Melanoma	Phage	(58)
TARP	FLRNFSLML	HLA-A*0201	Fab-D2	Fab-PE38	Breast, prostate	Phage	(75)
p53	LLGRNSFEV	HLA-A*0201	I3.M3-2A6	–	–	Hybridoma	(76)
p53	RMPEAAPPV	HLA-A*0201	T1-116C	IgG1	Breast	Hybridoma	(56)
p53	RMPEAAPPV	HLA-A*0201	T1-29D, T1-84C	IgG1, IgG2b	–	Hybridoma	(77)
p53	GLAPPQHLIRV	HLA-A*0201	T2-108A, T2-2A, T2-116A	IgG1, IgG2a, IgG1	–	Hybridoma	(77)
Tyrosinase	YMDGTMSQV	HLA-A*0201	TA2	Fab	Melanoma	Phage	(78)
p68	YLLPAIVHI	HLA-A*0201	RL6A	mIgG2a	Breast	Hybridoma	(79)
MIF	FLSELTQQL	HLA-A*0201	RL21A	IgG2a	Breast	Hybridoma	(80)
Proteinase 3	VLQELNVTV	HLA-A*0201	8F4	IgG2a	AML	Hybridoma	(81)
WT1	RMFPNAPYL	HLA-A*0201	ESK1	hIgG1	Mesothelioma, leukemia, ovarian, colon	Phage	(82)
WT1	RMFPNAPYL	HLA-A*0201	F2, F3	Fab	Leukemia	Phage	(59)
WT1	RMFPNAPYL	HLA-A*0201	Clone45	scFv	Leukemia	Phage	(83)
HA-1H	VLHDDLLEA	HLA-A*0201	#131	scFv, scFv-CAR	Leukemia	Phage	(84)
PRAME	ALYVDSLFFL	HLA-A*0201	Pr20	hIgG1	Leukemia, lymphoma, melanoma, breast, colon	Phage	(85)

*Published TCRm antibodies targeting cancer antigens are summarized*.

*PE38, 38 kDa immunotoxin, which is a truncated form of Pseudomonas exotoxin that can be conjugated to a TCRm Ab. sdAb, single domain antibody that has a single antigen binding domain originating from llama VHH. CAR, chimeric antigen receptor*.

### Production of TCRm Antibodies

T-cell receptor mimic antibodies are not as commonly available as traditional antibodies; this may be a consequence of the difficulty of their production in addition to the technology being less established. Recently, there has been an increase in the generation of TCRm antibodies targeting a variety of cancer or viral T-cell epitopes due to advances in the necessary technologies and techniques. TCRm antibodies have been produced either by immunization or by phage display, with both strategies presenting their respective pros and cons. One of the main limitations in the production of TCRm antibodies by both strategies was the correct refolding of recombinant peptide–MHC complexes and their purification (53). Recombinant peptide–MHC complexes are made by using bacterial expression to generate inclusion bodies containing the extracellular domains of the heavy chain of human leukocyte antigen (HLA) and β2-microglobulin. These are then refolded with the MHC-restricted peptide to generate correctly refolded monomers of high purity, in quantities that are sufficient for downstream applications. The correct refolding can be verified by structural and functional experiments, and the monomers can then be biotinylated for specificity and affinity characterization, and for antibody isolation (86–88).

Initially, TCRm antibodies were produced using hybridoma technology. The immunization methods used in these experiments limited the successful generation of TCRm antibodies. Antigen-presenting cells harboring immunogenic peptides in the groove of their MHC molecules were used as immunogens (89, 90). Obtaining TCRm antibodies of the correct specificity by employing this method yielded very few antibodies and many efforts proved to be unsuccessful (91). Since then, more successful attempts have been made by using recombinant peptide–MHC complexes, such as tetramers, in the immunization protocol, followed by high-throughput screening in order to isolate specific TCRm antibodies out of a pool of thousands of clones (62, 71, 81). This requires stable peptide–MHC-I binding and has resulted in the production of TCRm against tumor and viral T-cell epitopes.

While the traditional strategy for making TCRm antibodies is hybridoma technology, in the mid 1990s, it was shown that phage display technology could also be used to isolate antibodies (87). In this method, libraries of phage particles are generated, where each phage displays a unique antibody (a scFv or a Fab fragment) as a fusion protein on their surface. Each phage particle has the genes that encode the particular antibody that is expressed on its cell surface. Therefore, it is possible to select different phage particles by assessing whether they bind a target and thereby isolate the antibodies that have the desired specificity. The bound phages are then eluted and amplified in bacteria. Phage display technology has been used to isolate various TCRm antibodies against cancer antigens (63, 69, 92, 93).

Most TCRm antibodies published thus far have used phage display libraries for antibody production. Investigators argue that the main advantage of phage display is that it is efficient while being a relatively fast method (53). On the other hand, hybridoma technology is a relatively slower strategy, and it requires the immunogenic peptide and the MHC complex to bind with high affinity and form a very stable complex in order for the complex to persist throughout the immunization and *in vivo* IgG maturation. Nevertheless, the advantages of hybridoma technology include the isolation of antibodies that have a high affinity (in the low nanomolar range) for the peptide–MHC complex. This is due to the fact that antibodies undergo multiple antigen challenges and affinity maturation *in vivo*. Whereas affinities of TCRm antibodies produced through phage display tend to lie in the moderate nanomolar range (≈50–300 nM) and many require further *in vitro* affinity maturation (94, 95).

Furthermore, the antibodies produced through hybridoma technology are bivalent IgG isotype antibodies, whereas antibodies isolated using phage display are either scFv or Fab fragments (i.e. in the monovalent form with no Fc region). The Fc portion of the antibody is crucial in recruiting components of the immune system for cytotoxic effects mediated through ADCC and CDC. Antibodies in the monovalent form have reduced avidity (functional affinity) and increased turnover rates, which are undesirable when targeting epitopes that may be expressed at low densities, such as epitopes on tumor-associated peptide-MHC complexes (53). To circumvent this difficulty, further engineering can be undertaken to address these limitations. For example, scFv or Fab tetramers can be generated through biotinylation, thereby increasing their avidity or antibodies can be engineered to have a classical Fc region. On the other hand, monovalent antibody fragments are ideal for studies of epitope presentation and structure, as well as being used as the targeting moieties that deliver a conjugated toxin to target cells. One advantage over immunization of mice to generate antibodies (unless using those genetically engineered to have a human B-cell repertoire) is the possibility to generate fully human antibodies from display libraries.

### Considerations for Selecting TCRm Antibody Targets

The ideal target for a TCRm antibody would be a disease-specific peptide–MHC complex that is present at high density on the target cell surface while being absent from other normal cells. When considering TCRm antibodies against tumor targets, such peptides are most likely to arise from overexpressed proteins, which have a short half-life and, hence, a high turnover rate (96). Targeting an antigen with a functional role in tumor biology will also help avoid loss of the antigen under subsequent therapeutic selection pressure. The peptides must also have a high affinity for the patient’s MHC and form a stable complex that persists on the cell surface, allowing recognition by TCRm antibodies.

Antigens that could be promising therapeutic targets include peptides processed from mutated proteins, which are tumor specific, such as KRAS G12V/D or oncogenic fusion proteins (97, 98). Over-expressed genes, cancer testis antigens, and re-expressed oncofetal proteins are also potential tumor targets, for example, CEA and WT1 (99). The expression of these targets on normal healthy tissues must be considered when developing these as therapeutics (100). TCRm antibodies could also have use in targeting cells of the tumor microenvironment, such as regulatory T cells, tumor-associated macrophages, or cells with a role in angiogenesis (101, 102).

A key factor that needs to be considered when choosing a target antigen for TCRm antibody therapy includes the epitope expression on the cell surface. It is important to consider that it is the presentation of the epitope, and not expression of the antigen *per se*, that will determine the availability of antibody binding sites. Epitope density of TCRm antibody targets has been reported to be as low as 100–1,000 sites per cell, which is significantly lower than some epitope densities reported for traditional mAb cell surface targets at 20,000–500,000 sites per cell. Nevertheless TCRm can activate ADCC against low-density targets (82, 103, 104). Before being presented on the MHC molecule, the peptide undergoes various steps of processing from its original protein. Therefore, events at any of these steps could affect the epitope density observed at the cell surface, including the level of protein expression and its half-life, the peptide processing, the MHC levels, and the presentation of the peptide in the context of MHC at the cell surface. Proteins must be stable and translated in sufficient quantities to allow peptide processing, and it has been shown that proteins with shorter half-lives are more likely to be presented than ones with longer half-lives (105). Furthermore, it has been reported that tumors downregulate their surface MHC expression as an immune evasion mechanism, suggesting that such tumors will be less susceptible to TCRm therapy (106, 107). The possibility of this evasion mechanism must be considered when selecting both target antigens and disease indications.

### Target Epitope Discovery

Progress in our understanding of peptide processing and presentation on MHC has facilitated the discovery and evaluation of novel peptide–MHC epitopes. Initially, expression profiling was used to identify epitopes on tumor-associated antigens (TAAs) found on tumor cells—a process also called “direct immunology.” Using this method, the isolation of tumor-specific CTLs from melanoma patients led to the discovery of the first tumor-specific CTL epitope, which was encoded by the MAGE-1 gene. A cDNA library of the melanoma was generated, and melanoma-specific CTLs were used to identify the cDNA that encoded the CTL epitope (108, 109). Since this initial discovery, the use of direct immunology has led to the identification of other epitopes, including ones from the MAGE, BAGE, and GAGE families, as well as Melan-A/MART-1, tyrosinase, and gp100.

Bioinformatics techniques using algorithms to predict peptide binding to specific MHC molecules are often used to predict TAA epitopes. This process is known as “reverse immunology” and is a systematic method of identifying TAA epitopes from a defined antigen that has emerged from the recent progress in genome sequencing and *in silico* techniques. It involves a prediction phase, where potential epitopes are predicted *in silico* using algorithms. The prediction of epitopes is based on proteasome processing, binding to MHC and TAP translocation. This is followed by the validation phase, where the predicted epitopes must then be verified by MHC-I peptide binding assays or mass spectrometry to confirm that they are found on the cell surface (110).

There are a significant number of peptides that while being capable of binding MHC-I are either not presented on cancer cells or are altered, for example, by post-translational modification. Thus, there has been considerable interest in performing cancer HLA peptidome analysis to identify MHC-I bound peptides within both normal and malignant cells and tissues (111). In this approach, the HLA-complexes are immunoaffinity purified, the bound peptides are isolated and then analyzed by mass spectrometry. By comparing the MHC-I bound peptides in normal and diseased tissues, it is possible to prioritize those epitopes that are most suitable for therapy. Interestingly a meta-analysis of the HLA peptidomes from 83 mass spectrometry-based datasets from four major hematological malignancies found very few common “pan-leukemia” epitopes and these exhibited low presentation frequencies within each cohort of patients (112). Thus, in hematological malignancy, the epitopes selected for therapy are likely to be disease specific and, thus, multiple TCRm antibodies will be needed to exploit this therapeutic approach.

### TCRm Antibodies and TCR-Based Therapies

Both TCRm antibodies and recombinant TCRs can bind MHC-I presented peptides. Traditionally, those employing TCR-based therapies have compared their technology to the desirable qualities of antibodies but commented on the inability of antibodies to target intracellular antigens. Those generating TCRm/TCR-like antibodies have promoted antibodies having higher affinity and specificity than TCRs (82, 113) and an easier development route and lower cost than TCR-targeted cellular therapies. However, advances in the engineering and production of soluble high-affinity TCRs and the production of TCRm antibodies have now made these approaches much more interchangeable.

T-cell receptor mimic antibodies can be used in place of a TCR as the targeting moiety for cellular therapies, such as CAR T cells (59, 60). Alternatively, a TCR can be fused to an Ig Fc region to enable TCR-directed antibody-dependent cytotoxicity (114). ImmTACs (immune-mobilizing monoclonal TCRs against cancer) are engineered high-affinity soluble TCRs bispecifically linked to anti-CD3 that can drive an anti-tumor T-cell response (115). Some studies have reported that the orientation of binding is similar for TCRs and TCRm antibodies, with both binding their peptide–MHC target in a diagonal orientation (116, 117). TCRm antibodies can also bind in additional conformations, gaining access to epitope regions that are not naturally targeted by TCRs (94, 118).

It seems likely that both TCRm antibodies and TCRs will be used to effectively target intracellular antigens using both soluble drugs and cellular therapies. The specificity of binding of high-affinity TCRs and TCRm antibodies to the target peptide presented by MHC-I is likely to be a crucial determinant of the suitability of individual reagents for therapy. Comparative studies using TCRs to the tumor-associated antigen survivin effectively highlighted the importance of specific peptide binding. High-affinity TCRs against a survivin peptide presented by HLA-A2 isolated from an allogeneic HLA-mismatched TCR repertoire lacked the ability to distinguish high levels on tumor cells from low expression in normal tissues. This included activated T cells, leading to fratricide when the engineered T cells targeted each other for destruction. However, an autologously derived TCR to the same survivin peptide targeted tumor cells but did not cause fratricidal toxicity (119). Molecular modeling of TCR–peptide–HLA complexes and alanine scanning of the survivin peptide demonstrated that maximal peptide recognition was critical for TCR selectivity for tumor cells. Thus, the specificity of the peptide–MHC binder could be as critical as the choice of target peptide.

### Future Directions for TCRm Antibodies

T-cell receptor mimic antibodies have not yet entered the clinic, although Novartis have partnered with Eureka Therapeutics and Memorial Sloan Kettering Cancer Center to develop their ESK1 TCRm targeting WT1. Several key factors have the potential to improve the development of TCRm antibodies further with the prospect of undertaking clinical studies and ultimately establishing them as cancer therapeutics. These include epitope expression, production methodology, specificity validation and mechanism of action. It is also important to consider that TCRm antibodies may represent theranostics, combining diagnostic utility to determine target epitope presentation and therapeutic activity within a single agent.

The low epitope density of peptide–MHC complexes on the cell surface poses some limitations for TCRm antibody-based therapy. This might be addressed by choosing target epitopes that do not have low cell surface expression, by increasing MHC-I expression in tumors, by making TCRm antibodies more sensitive to low density epitopes or by choosing effector mechanisms that do not require high epitope density for cytotoxicity.

High-affinity, peptide-specific TCRm antibodies have proven difficult to produce in large numbers by either traditional phage or hybridoma approaches. Enhanced display technologies, particularly those capable of isolating fully human antibodies within a short period of time, offer some exciting opportunities to accelerate future TCRm antibody discovery. Having a wider array of antibodies for characterization will improve the chances of identifying those with the necessary affinity and specificity for further development.

It is crucial to ensure that the TCRm antibody does not recognize the MHC-I alone, as this molecule is found on most nucleated cells. Therefore, the TCRm antibody must be specific for the peptide–MHC complex, which also highlights that it should not cross-react with other processed peptides. As the TCRm antibody recognizes only few amino acid residues in the peptide, it will be crucial to assess which other processed peptides possess the same amino acids at those positions and whether there would be any risk of cross-reactivity. The importance of this is exemplified in a clinical trial of an affinity-enhanced TCR, which targeted a MAGE-A3 epitope (120). Following administration of the therapy, it was discovered that the TCR also recognized an epitope on the unrelated protein titin that is expressed in cardiac tissue. The cardiac toxicity led to two patient deaths. This cross-reactivity was not observed in normal tissue screening and the titin peptide was not conserved in mice. However, a limitation of *in silico* screening by amino acid substitution is that it may identify a wider variety of potentially cross-reactive peptides than can be functionally evaluated. Furthermore, even potentially cross-reactive peptides shown to bind MHC-I in T2 presentation assays may not be processed endogenously or presented on the cell surface in normal tissues.

One of the key limitations of TCRm antibody therapy is the MHC-restricted nature of the therapy—although this is crucial to enable the recognition of intracellular proteins. Most studies to date focus on the HLA-A*0201 haplotype, which is prevalent in up to 40% of Caucasians, and in up to 20% of populations of different ethnicities, which covers a large proportion of the world’s population. There are other dominant HLA alleles worldwide, including HLA-A*2402 and HLA-A*1101 in Oriental populations. Although TCRm antibodies are HLA-restricted, it has been proposed that antibodies to three HLA alleles for a particular target antigen would cover >96% of the world’s population (53). Structural analysis of the TCRm antibody ESK1 shows that it binds multiple HLA-A*02 variants and not only the HLA-A*0201 subtype, which it is designed to target (121). This is due to the fact that ESK1 binds a portion of the MHC molecule that is conserved among the various HLA-A2 subtypes, thereby suggesting that the certain TCRm could target a larger population of patients with a variety of HLA subtypes. In addition, designing TCRm antibodies that target different antigens or different epitopes on the same antigen and using a combination of these as a therapeutic regimen could increase the chances of successful tumor eradication and minimize escape variants.

The manufacture and regulatory approval pathways for TCRm antibodies are likely to have similarities to that for classical monoclonal antibodies and share commonalities with TCR-based therapies. The latter being the lack of availability of suitable animal models to study agents targeting a dual epitope where potentially neither the MHC-I or target peptide is conserved. One opportunity potentially available for TCRm antibodies would be to use *in vivo* imaging studies to study the biodistribution of a subtherapeutic dose of the TCRm antibody in early clinical safety studies.

## Conclusion

The generation of antibodies that can target intracellular antigens offers an unparalleled opportunity to expand the repertoire of therapeutic antibodies that are available to treat human disease. When coupled with advances in genomic sequencing technologies, proteomic investigations and the increasing numbers of antibodies being made available to the research community, new disease-related proteins and their variants (post translational modifications, splice variants, mutations, etc.) that are suitable for antibody targeting will continue to be identified. Further developments in the production technology, delivery, and regulatory approval pathways for antibodies targeting intracellular antigens should also contribute to the introduction of many new exciting antibodies into the clinic in the future.

## Author Contributions

All authors contributed to drafting, revising, and approving the final article.

## Conflict of Interest Statement

AB and DL are inventors, and IT is a contributor, on a patent application describing the production and characterization of TCR mimic antibodies targeting p53:HLA-A2.
